# When high hopes meet low action: identifying the “Ambitious Procrastinator” profile in the physical activity intention-behavior gap and its mental health toll

**DOI:** 10.3389/fpsyg.2026.1804409

**Published:** 2026-03-30

**Authors:** Xishan Liu, Peijun Wei

**Affiliations:** 1 Nanyang Normal University, Nanyang, Henan, China; 2 Qilu Institute of Technology, Jinan, Shandong, China

**Keywords:** intention-behavior gap, latent profileanalysis, mental health, physical activity, post-pandemic adjustment, university students

## Abstract

**Background:**

University students exhibit high rates of mental health problems alongside a significant gap between their physical activity (PA) intentions and actual behavior. To understand the psychological heterogeneity within this intention-behavior gap (IBG) in high-pressure academic environments, a person-centered approach is essential. The present study aimed to identify distinct psychological profiles of students based on key self-regulatory constructs related to PA and to examine how these profiles longitudinally predict changes in mental health over an academic semester.

**Methods:**

A two-wave longitudinal survey was conducted with a cohort of 850 university students during the post-pandemic return to campus life, situated within a high-achieving Chinese higher education context. At baseline (T1), PA intention, action and coping planning, self-efficacy, maladaptive perfectionism, and procrastination were measured. At both T1 and the end of the semester (T2), PA behavior (IPAQ-SF) and mental health outcomes, including depression (PHQ-9), anxiety (GAD-7), and academic burnout (SBI) were assessed. Latent Profile Analysis (LPA) was employed to identify distinct profiles from the T1 psychological data. Longitudinal regression models were then used to test the predictive validity of these profiles on T2 mental health, controlling for T1 baseline mental health, demographic covariates, and critically, T1 baseline PA behavior.

**Results:**

LPA revealed four distinct profiles: “Effective Planners” (25.0%), “Ambitious Procrastinators” (30.0%), “Cautious Doers” (24.9%), and “Indifferent & Sedentary” (20.1%). The “Ambitious Procrastinators” exhibited the largest intention-behavior gap. Even after controlling for baseline PA behavior, membership in this profile significantly predicted greater increases in depression ( β=0.16 , p<0.001 ), anxiety ( β=0.14 , p=0.002 ), and academic burnout ( β=0.20 , p<0.001 ) at T2, relative to the “Effective Planners.”

**Conclusion:**

The physical activity intention-behavior gap is not a monolithic phenomenon, and the “Ambitious Procrastinators” represent a particularly vulnerable subgroup. Findings suggest that university wellness programs should move beyond generic motivational campaigns and instead deliver tailored, skill-based interventions**, such as specific cognitive restructuring and behavioral activation, **targeting the specific self-regulatory deficits of these high-risk students.

## 1 Introduction

Higher education marks a critical developmental period characterized by profound intellectual, social, and emotional transitions. This period, however, is increasingly defined by a dual crisis on university campuses worldwide: deteriorating mental health and pervasive physical inactivity. Epidemiological data underscore this urgency; the WHO World Mental Health Surveys International College Student Project indicates that approximately 35% of university freshmen meet the criteria for at least one lifetime mental disorder, with depression and anxiety being the most prevalent (Auerbach et al., 2018).

Critically, in the wake of the COVID-19 pandemic, this vulnerability has been exacerbated. Stockwell et al. (2021) indicate that the prolonged shift to online learning has fostered a “digital-sedentary” lifestyle. Even after the resumption of in-person classes, researchers have observed a phenomenon of “lifestyle inertia,” where the erosion of external regulatory structures (e.g., mandatory sports schedules) during lockdowns has placed a heavier burden on students’ internal self-regulation (Cheval et al., 2021). This volitional deficit is particularly acute in East Asian higher education settings, such as in China, where an intense culture of academic “involution” (Neijuan) imposes chronic stress and cultivates high perfectionistic standards, further depleting the limited cognitive resources required for health-promoting behaviors. This creates a potential deleterious feedback loop wherein physical inactivity exacerbates poor mental health.

Concurrently, despite physical activity (PA) being a highly effective and accessible strategy for mitigating stress and improving mood, the majority of students fail to meet global PA guidelines. The transition from structured high school environments to the autonomy of university life often precipitates a steep decline in PA. This decline is particularly concerning in the post-pandemic era, where students often experience delayed physiological and psychological sequelae, including reduced physical fitness and increased sedentary behavior patterns. This creates a potential deleterious feedback loop, wherein physical inactivity exacerbates poor mental health, which in turn diminishes motivation for adaptive behaviors.

Within health psychology, the discrepancy between “wanting” and “doing” is known as the “intention-behavior gap” (IBG). Early rational models, such as the Theory of Planned Behavior (TPB), identified intention as the proximal determinant of behavior but were criticized for neglecting the post-intentional phase (Sniehotta et al., 2005). While Rhodes and de Bruijn (2013) famously quantified this gap, noting that nearly half of “intenders” fail to act, more recent frameworks, such as the Multi-Process Action Control (M-PAC) approach (Rhodes, 2017), posit that translating intention into action is not merely a linear process but a complex interplay between reflective regulation and reflexive tendencies. According to this evolving perspective, the failure to act often stems from a lack of “volitional shielding”—the cognitive ability to protect intentions from competing distractions and fatigue (Rhodes and Rebar, 2017). Consequently, motivation remains a necessary but insufficient condition for action.

Traditional variable-centered research often treats this gap as a uniform failure. However, contemporary theory suggests that the “volitional shielding” failure can stem from different sources: a lack of regulatory skills (e.g., planning) or an excess of regulatory liabilities (e.g., procrastination). A person-centered approach (Latent Profile Analysis) is therefore essential to capture how these specific “configurations” of adaptive skills and maladaptive traits co-occur within individuals to inhibit behavior.

## 2 Literature review and hypothesis development

### 2.1 The intention-behavior gap in a post-pandemic context

Social-cognitive models, most notably the Theory of Planned Behavior, have long posited intention as the proximal determinant of human behavior. Yet, the translation of intention into action is frequently imperfect. While early research established the magnitude of this gap (Rhodes and de Bruijn, 2013), more recent frameworks, such as the Multi-Process Action Control (M-PAC) approach (Rhodes, 2017), emphasize that behavioral translation is not a linear process but a complex interplay between reflective regulation and reflexive tendencies. As discussed, the high rate of failure among “intenders” underscores the necessity of moving beyond purely motivational paradigms.

This gap appears to be widening among university students in the post-pandemic era. Recent evidence suggests that the prolonged shift to online learning fostered a “digital-sedentary” lifestyle (Stockwell et al., 2021). Even after the resumption of in-person classes, the erosion of external regulatory structures (e.g., mandatory sports schedules) has placed a heavier burden on students’ internal self-regulation. Consequently, interventions focused solely on motivating students are increasingly insufficient.

### 2.2 Integrated self-regulatory framework and variable selection

To systematically investigate the psychological heterogeneity of the IBG, the present study adopts an “Integrated Self-Regulatory Framework” that synthesizes the Health Action Process Approach (HAPA) (Schwarzer, 2008) with the theory of Self-Regulation Failure (Sirois and Pychyl, 2013). This framework juxtaposes “Volitional Competence” against “Volitional Liability.”

First, regarding Volitional Competence, HAPA posits that the transition from intention to action requires specific self-regulatory skills. We selected Action Planning (specifying “when, where, and how”) and Coping Planning (anticipating barriers) as the primary indicators, as these constructs represent the conscious cognitive strategies necessary to initiate behavior. We also included Self-Efficacy, which serves as the foundational belief supporting these planning efforts (Renner and Schwarzer, 2003).

Second, regarding Volitional Liability, we acknowledge that behavior is also influenced by automatic processes such as habit (Rhodes, 2017). However, the present study is situated in a specific “post-pandemic” context characterized by “Habit Disruption” (Wood et al., 2005). Following prolonged lockdowns, students’ automatic behavioral loops were broken. Theory suggests that when habits are disrupted, behavior regulation reverts from automaticity to effortful, conscious self-control (Lally and Gardner, 2013). In this context, the primary barrier to action is not the lack of habit strength, but the presence of dispositional “saboteurs” that deplete the cognitive resources needed for conscious regulation. Therefore, we selected Academic Procrastination and Maladaptive Perfectionism (Concern over Mistakes) as key indicators of regulatory liability. These traits represent specific self-regulatory failures that directly compete with the limited cognitive resources required for health behavior execution (Sirois and Pychyl, 2013).

### 2.3 The link to mental health: a self-discrepancy perspective

While physical inactivity is a known risk factor for poor mental health, the psychological experience of the intention-behavior gap may exert a unique pathogenic effect Higgins (1987). Self-Discrepancy Theory provides the theoretical mechanism for this link. The theory posits that individuals hold an “Ideal Self” (represented by their high PA intention) and an “Actual Self” (represented by their sedentary behavior). When a significant discrepancy exists between these selves, it induces distinct “dejection-related emotions,” such as disappointment, dissatisfaction, and depression. Therefore, we argue that students who are highly motivated but fail to act (the “gap” group) suffer from a chronic state of self-discrepancy, which may be psychologically more taxing than being unmotivated (where no discrepancy exists).

### 2.4 Hypotheses

Based on this integrated framework, three hypotheses were formulated:

*H1*: LPA will reveal multiple, heterogeneous profiles, challenging a simplistic “adaptive vs. maladaptive” dichotomy.

*H2*: A distinct “discordant” profile will emerge, characterized by a pattern of “Intention-Capacity Dissociation”—specifically, high PA intention and perfectionism (high standards) undermined by low planning capabilities and high procrastination (low regulatory capacity).

*H3*: Based on Self-Discrepancy Theory, membership in this discordant profile will longitudinally predict the greatest increases in mental health problems (depression, anxiety, burnout) at T2, due to the psychological distress of failing to enact valued goals.

## 3 Methods

### 3.1 Participants and procedure

The present study employed a two-wave longitudinal design, with data collected during the Fall semester of the 2024–2025 academic year. The initial assessment (T1) was administered in September 2024 (Weeks 1–3), marking the students’ full return to stable offline campus life following the previous years’ pandemic-related disruptions. The follow-up assessment (T2) took place at the end of the semester in December 2024 (Weeks 12–14).

Participants were recruited from Nanyang Normal University, a comprehensive university in Henan Province, Central China. A convenience sample of full-time undergraduate and graduate students was recruited via university-wide email listservs, course announcements, and digital campus posters across diverse faculties (Arts, Sciences, and Engineering) to ensure a representative cross-section of the student body. This demographic is particularly relevant, as students in such comprehensive Chinese universities frequently experience intense academic pressure driven by the pervasive “involution” (Neijuan) culture, making them highly susceptible to perfectionistic concerns and burnout. Eligibility criteria included being 18 years or older and enrolled in a full-time degree program.

At T1, 850 students completed the initial online survey, which was administered via the secure platform “QuestionPro” and took approximately 25 min. This survey included all demographic, T1 psychological, baseline PA, and baseline mental health measures. At T2, participants who had consented to follow-up were sent a link to the second survey, which reassessed PA and mental health. Of the initial 850 participants, 792 completed the T2 survey, yielding a high longitudinal retention rate of 93.2%. To examine potential attrition bias, independent samples t-tests were conducted to compare T2 completers ( n=792 ) and non-completers ( n=58 ) on key T1 variables. Results indicated no significant differences in baseline PA behavior ( t=0.45,p=0.65 ), PA intention ( t=0.82,p=0.41 ), baseline depression ( t=1.03,p=0.30 ), baseline anxiety ( t=0.98,p=0.33 ), or academic burnout ( t=1.12,p=0.26 ). These findings suggest that data were likely missing at random and attrition was not driven by the variables of interest.

Regarding ethical incentives, undergraduate participants received partial course credit. To ensure procedural equity, graduate students (who do not receive course credit) were offered small monetary compensation (20 CNY for T1, 30 CNY for T2) of equivalent value. Crucially, to adhere to ethical standards and ensure non-coercion, students who chose not to participate in the research were offered an equivalent, non-research alternative assignment (e.g., attending a 30-min wellness lecture or submitting a brief reflection paper) to earn the same amount of credit. This ensured that participation was strictly voluntary.

Data security and anonymity were strictly maintained. All data were stored on encrypted servers accessible only to the principal investigator, and identifiable information was removed prior to analysis. The study protocol was approved by the Institutional Review Board of the School of Physical Education, Nanyang Normal University (IRB Approval No: 2024-075), and all participants provided electronic informed consent prior to participation.

### 3.2 Measures

All instruments were administered in Mandarin Chinese, using validated versions with established psychometric properties in Chinese university populations. Unless otherwise specified, psychological constructs were measured using 7-point Likert scales ranging from 1 (Strongly Disagree) to 7 (Strongly Agree).

#### 3.2.1 PA-related self-regulation constructs (T1)

The assessment of PA-related self-regulation began with PA Intention, measured using two items assessing the strength of the participant’s commitment to exercise (e.g., “I plan to engage in at least 3 sessions of moderate-intensity physical activity per week for the next month”; α=0.88 ; Ajzen, 1991). Following this, Action and Coping Planning were evaluated using the 6-item scale from Schwarzer (2008) ( α=0.91 ). Finally, PA Self-Efficacy was measured with a 5-item scale adapted from Renner and Schwarzer (2003) ( α=0.89 ). It is noted that while PA intention items referred to “the next month,” PA behavior was assessed over “the past week”. While this temporal mismatch is common in IBG research to capture general motivational orientation versus recent behavior, it is acknowledged as a measurement limitation.

#### 3.2.2 Volitional barriers (T1)

To assess psychological barriers to action, we focused on dispositional traits that generalize to health behaviors. Perfectionism was assessed using the “Concern over Mistakes” subscale of the Frost Multidimensional Perfectionism Scale (FMPS) (Frost et al., 1990) ( α=0.86 ). While the FMPS contains multiple dimensions, “Concern over Mistakes” was specifically selected as it represents the core maladaptive element of perfectionism most strongly linked to behavioral paralysis and avoidance in health contexts (Stoeber and Otto, 2006). The Chinese version of this subscale has demonstrated good construct validity and reliability in Chinese university samples (Zi and Zhou, 2006).

#### 3.2.3 Physical activity behavior (T1 & T2)

Physical activity was assessed at both time points using the International Physical Activity Questionnaire-Short Form (IPAQ-SF; Macfarlane et al., 2007). Following official scoring protocols, data were cleaned and truncated, and total weekly PA was calculated as MET-minutes/week.

#### 3.2.4 Mental health outcomes (T1 & T2)

Depression was assessed using the PHQ-9 ( α>0.88 ; Spitzer et al., 1999). Anxiety was measured using the GAD-7 ( α>0.87 ; Spitzer et al., 2006). Finally, Academic Burnout was assessed using the Chinese version of the School Burnout Inventory (SBI) (Salmela-Aro et al., 2009). This 9-item instrument measures Exhaustion, Cynicism, and Sense of Inadequacy. The scale demonstrated excellent internal consistency ( α>0.88 ).

### 3.3 Statistical analysis

All statistical analyses were conducted using SPSS 26.0 and Mplus 8.8. First, data were screened for missingness, which was minimal (<2% on any item) and handled with pairwise deletion for correlational analyses to maximize data utility in preliminary descriptions, and Full Information Maximum Likelihood (FIML) in Mplus to utilize all available data points for robust model estimation regarding the LPA. Descriptive statistics and a Pearson bivariate correlation matrix for all key T1 variables were computed.

The primary analysis, Latent Profile Analysis (LPA), was conducted in Mplus 8.8 to address H1 and H2. The five T1 psychological constructs (intention, planning, self-efficacy, perfectionism, procrastination) were used as indicators. To ensure model stability and avoid local maxima, models were estimated using 500 random sets of starting values and 50 final stage optimizations. The selection of the optimal number of profiles was guided by information criteria (BIC, aBIC), the Lo–Mendell–Rubin Likelihood Ratio Test (LMR-LRT), and theoretical interpretability. Although information criteria continued to decrease for 5- and 6-profile solutions, these models were rejected due to the emergence of small profiles (<5% of sample) that lacked theoretical distinctiveness and stability. Once the optimal solution was identified, participants were assigned to their most likely profile based on posterior probabilities.

To test our longitudinal hypothesis (H3), a series of parallel multiple linear regression models were conducted in SPSS. It is acknowledged that this “classify-analyze” approach (assigning participants to their most likely profile) does not account for classification uncertainty, which may bias standard errors. However, given the high entropy (0.89) of our solution, regression analysis was deemed appropriate. To rigorously verify the robustness of these findings and address the limitations of the “classify-analyze” approach, a sensitivity analysis was subsequently conducted using the Bolck-Croon-Hagenaars (BCH) three-step method in Mplus, which preserves classification uncertainty when estimating auxiliary models. Three separate models predicted T2 Depression, T2 Anxiety, and T2 Academic Burnout. In each model, the primary predictors were dummy-coded variables representing profile membership. Critically, to rigorously test the predictive validity of profiles on T2 mental health change, all regression models controlled for T1 baseline mental health levels, demographic covariates (age, gender, academic year), and T1 baseline PA behavior (MET-min/week). Controlling for baseline PA is essential to ensure that profile effects reflect psychological mechanisms rather than simply the carry-over effects of prior physical inactivity. To further clarify the predictive utility of the profile memberships, changes in explained variance ( $\Delta R^{2}$ ) were extracted for each hierarchical regression step.

## 4 Results

### 4.1 Descriptive statistics and bivariate correlations

Descriptive statistics and bivariate correlations for all key T1 variables are presented in Table 1. All T1 measures exhibited good to excellent internal consistency (Cronbach’s α=0.86 to 0.91). As expected, the adaptive self-regulatory constructs (intention, planning, self-efficacy) were moderately to strongly inter-correlated ( rs=0.48 to 0 .68,p<0.001 ). Procrastination showed strong negative correlations with planning ( r=−0.52,p<0.001 ) and self-efficacy ( r=−0.48,p<0.001 ), supporting its role as an antagonist to self-regulation. Maladaptive perfectionism (concern over mistakes) was positively associated with both procrastination ( r=0.31,p<0.001 ) and PA intention ( r=0.25,p<0.001 ).

**Table 1:** Descriptive statistics and bivariate correlations for key variables at T1 ( N=850 ).

Variable	M (SD)	1	2	3	4	5	6	7	8	9	10	11
1. Age	20.35 (1.88)	—										
2. Gender (1 = M)	0.36 (0.48)	0.03	—									
3. PA intention	5.20 (1.45)	0.05	0.08*	(0.88)								
4. PA planning	4.15 (1.60)	0.08*	0.05	0.55**	(0.91)							
5. PA self-efficacy	4.30 (1.55)	0.04	0.11*	0.48**	0.68**	(0.89)						
6. Perfectionism	3.90 (1.30)	0.06	0.02	0.25**	−0.10*	0.15*	(0.86)					
7. Procrastination	4.45 (1.40)	0.05	0.09*	−0.52**	−0.48**	0.31**	(0.90)					
8. T1 PA behavior	1,405 (950)	0.04	0.15**	0.35**	0.41**	0.40**	−0.18**	−0.33**	—			
9. T1 depression	9.40 (4.50)	0.02	−0.06*	0.20**	0.35**	−0.38**	0.30**	0.45**	−0.28**	(0.88)		
10. T1 anxiety	8.80 (4.20)	0.00	−0.05	0.18**	−0.31**	−0.33**	0.33**	0.42**	−0.25**	0.75**	(0.87)	
11. T1 burnout	3.20 (1.40)	0.03	−0.01	0.22**	−0.38**	−0.40**	0.35**	0.50**	−0.30**	0.70**	0.72**	(0.92)

M (SD) = Mean (Standard Deviation). Cronbach’s α coefficients are on the diagonal in parentheses. PA = physical activity; T1 PA behavior is in MET-minutes/week. perfectionism refers to the concern over mistakes subscale. * p<0.05 , ** p<0.01 .

### 4.2 Latent profile analysis and profile identification

To address H1 and H2, we conducted an LPA on the five standardized T1 psychological indicators. The fit indices for models with one to six profiles are presented in Table 2.

**Table 2:** Model fit indices for latent profile analysis solutions.

Model	AIC	BIC	ABIC	Entropy	LMR-LRT *p*-value	Smallest class (%)
1-Profile	11520.1	11565.3	11538.5	—	—	100
2-Profile	10980.4	11048.2	11005.1	0.81	<0.001	38.5
3-Profile	10650.7	10741.0	10681.6	0.86	<0.001	24.0
**4-Profile**	**10410.2**	**10523.1**	**10447.3**	**0.89**	**<0.001**	**20.1**
5-Profile	10355.6	10491.0	10398.8	0.85	0.182	8.2
6-Profile	10310.9	10468.9	10360.3	0.87	0.219	5.5

The selected model is in bold.

As shown in Table 2, the 4-profile solution provided the best fit to the data, yielding high Entropy (0.89) and statistically significant LMR-LRT ( p<0.001 ), whereas the 5-profile solution offered no significant improvement. Furthermore, the average posterior probabilities for the most likely profile membership ranged from 0.86 to 0.94, indicating high classification accuracy and clear separation between the identified subgroups.

**Figure 1:**
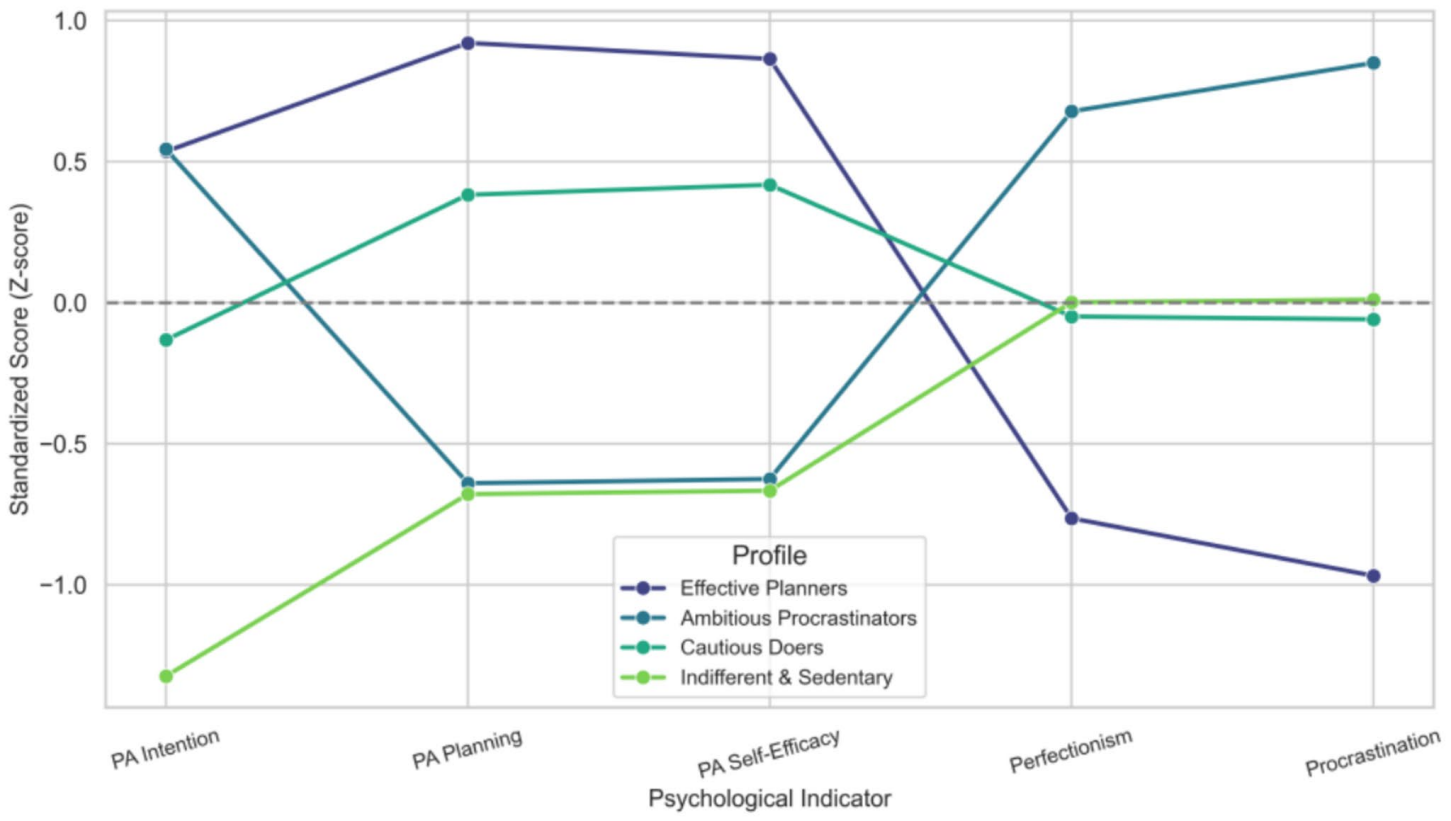
Psychological profiles of the physical activity intention-behavior gap (*Z*-scores).

As visually depicted in Figure 1, the analysis identified four distinct profiles. The first, labeled “Effective Planners” (25.0%), represented the most adaptive group, characterized by high intention, planning, and self-efficacy alongside low procrastination. In contrast, the largest group, termed “Ambitious Procrastinators” (30.0%), exhibited a paradoxical pattern of the highest intention and perfectionism but critically low planning and self-efficacy combined with high procrastination. The remaining profiles included “Cautious Doers” (24.9%), who showed moderate intention but relatively high planning and self-efficacy, and the “Indifferent & Sedentary” group (20.1%), defined by low levels across all measured constructs.

### 4.3 Profile characteristics and validation

Post-hoc analyses were conducted to validate the profiles against demographic and baseline variables (Table 3). No significant differences emerged across the profiles for age, F(3,846)=1.09,p=0.350 . Crucially, to address potential concerns regarding sample heterogeneity, sensitivity analyses were conducted. Chi-square tests revealed no significant differences in profile distribution between undergraduate and graduate students ( $\chi^{2}(3)=3.85,p=0.28$ ), nor between male and female participants ( $\chi^{2}(3)=2.11,p=0.55$ ). This indicates that the identified profiles are robust patterns that appear consistently across different education levels and genders.

**Table 3:** Comparison of demographic, behavioral, and health characteristics across the four latent profiles at T1 ( N=850 ).

Variable	P1: effective planners (*n* = 213)	P2: ambitious procrastinators (*n* = 255)	P3: cautious doers (*n* = 212)	P4: indifferent & sedentary (*n* = 170)	F/χ2	*p*
Demographics						
Age, M (SD)	20.4 (1.9)	20.3 (1.8)	20.5 (2.0)	20.2 (1.9)	1.09	0.350
Gender (% Female)	63.8%	65.1%	66.0%	60.6%	2.11	0.550
T1 psychological Z-scores						
PA intention	1.05	1.10	0.05	−1.65	315.4	<0.001
PA planning	1.22	−0.78	0.53	−0.97	401.2	<0.001
PA self-efficacy	1.13	−0.77	0.55	−0.90	388.7	<0.001
Perfectionism	−0.85	0.81	0.00	−0.04	102.6	<0.001
Procrastination	−1.32	1.07	−0.11	−0.04	350.1	<0.001
T1 behavior & health, M (SD)						
PA behavior (MET-min)	2,150 (880)^a^	950 (550)^c^	1,650 (710)^b^	810 (520)^c^	112.9	<0.001
Depression (PHQ-9)	5.10 (2.5)a	11.20 (4.1)^c^	8.90 (3.8)^b^	10.90 (4.4)^c^	88.4	<0.001
Anxiety (GAD-7)	4.50 (2.1)^a^	10.50 (3.9)^c^	8.10 (3.5)^b^	9.90 (4.0)^c^	79.2	<0.001
Burnout (SBI)	2.15 (1.1)^a^	3.80 (1.4)^c^	3.10 (1.2)^b^	3.65 (1.3)^c^	65.3	<0.001

Z-scores are based on the full sample. Means with different superscripts (a, b, c) are significantly different at p<0.05 (Tukey HSD).

**Figure 2:**
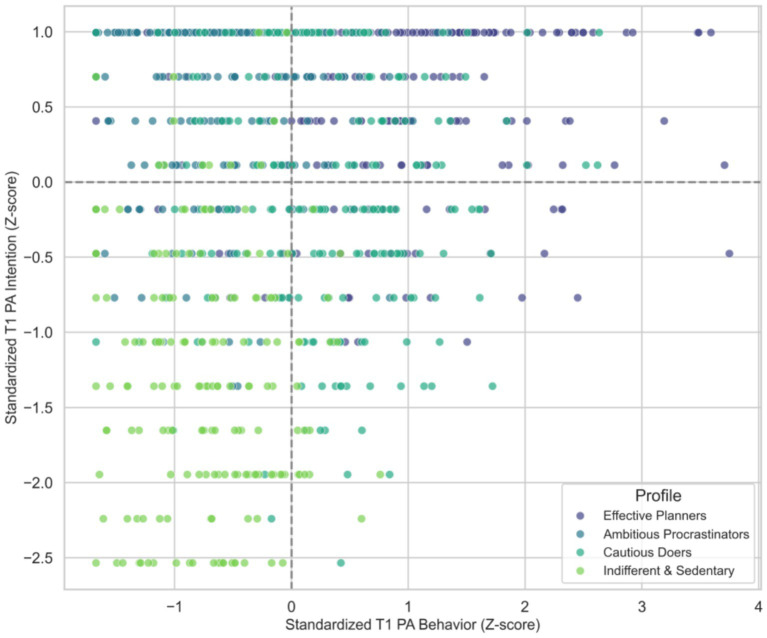
Visualization of the intention-behavior gap by latent profile.

However, large and significant differences were found for T1 PA behavior and mental health. As illustrated in Figure 2, the “Ambitious Procrastinators” reported PA levels ( M=950 ) that were significantly lower than “Effective Planners” and not statistically different from the “Indifferent & Sedentary” group ( M=810 ), despite having the highest intentions. This visualization confirms the presence of a severe intention-behavior gap.

### 4.4 Longitudinal prediction of T2 mental health

Our primary longitudinal analysis (H3) examined whether profile membership predicted changes in T2 mental health outcomes. To rigorously test this, hierarchical regression models controlled for demographic covariates (age, gender, academic year), baseline mental health, and critically, T1 PA behavior in Step 1, before entering the profile membership variables in Step 2.

The results (Table 4) strongly supported H3. The inclusion of profile memberships contributed a significant proportion of incremental variance across all three mental health outcomes (Depression: $\Delta R^{2}=0.04,p<0.001$ ; Anxiety: $\Delta R^{2}=0.03,p<0.001$ ; Burnout: $\Delta R^{2}=0.05,p<0.001$ ). Even after accounting for the protective effects of prior physical activity, membership in the “Ambitious Procrastinators” profile remained a significant predictor of mental health decline.

**Table 4:** Longitudinal regression models predicting T2 mental health outcomes from profile membership ( N=792 ).

Outcome variable (T2) & predictor	B	SE	β	p	95% CI
T2 Depression (PHQ-9)					
Model $R^{2}=0.49,F(7,784)=108.5,p<0.001$					
$\Delta R^{2}$ (Profile block) = 0.04, p<0.001					
T1 PHQ-9 (covariate)	0.58	0.04	0.56	<0.001	[0.50, 0.66]
T1 PA behavior (covariate)	−0.001	0.00	−0.09	0.003	[−0.002, 0.00]
(Ref: P1 effective planners)					
P2: ambitious procrastinators	1.52	0.16	0.34	<0.001	[0.85, 2.19]
P3: cautious doers	0.35	0.35	0.03	0.315	[−0.34, 1.04]
P4: indifferent & sedentary	1.10	0.11	0.38	0.004	[0.35, 1.85]
T2 anxiety (GAD-7)					
Model $R^{2}=0.52,F\;(7,784)=121.2,p<0.001$					
$\Delta R^{2}$ (Profile block) = 0.03, p<0.001					
T1 GAD-7 (covariate)	0.61	0.04	0.59	<0.001	[0.53, 0.69]
T1 PA behavior (covariate)	−0.001	0.00	−0.08	0.005	[−0.002, 0.00]
(Ref: P1 effective planners)					
P2: ambitious procrastinators	1.21	0.14	0.30	0.002	[0.62, 1.80]
P3: cautious doers	0.28	0.32	0.03	0.380	[−0.35, 0.91]
P4: indifferent & sedentary	0.95	0.34	0.10	0.006	[0.28, 1.62]
T2 academic burnout (SBI)					
Model $R^{2}=0.56,F\;(7,784)=142.6,p<0.001$					
$\Delta R^{2}$ (Profile block) = 0.05, p<0.001					
T1 SBI (covariate)	0.55	0.03	0.52	<0.001	[0.49, 0.61]
T1 PA behavior (covariate)	−0.000	0.00	−0.07	0.008	[−0.001, 0.00]
(Ref: P1 effective planners)					
P2: ambitious procrastinators	1.44	0.27	0.20	<0.001	[0.91, 1.97]
P3: cautious doers	0.22	0.28	0.03	0.435	[−0.33, 0.77]
P4: indifferent & sedentary	1.15	0.14	0.30	0.001	[0.56, 1.74]

B = Unstandardized coefficient; SE = Standard Error; β = Standardized coefficient; CI = Confidence Interval. All models controlled for age, gender, academic year, and T1 PA behavior (MET-min/week). Coefficients for demographic covariates are provided in Supplementary Table S1. Estimated $\Delta R^{2}$ reflects the unique variance explained by adding the profile dummy variables after controlling for baseline metrics.

When predicting T2 Depression, after controlling for T1 depression, T1 PA behavior, age, gender, and academic year, profile membership remained a significant predictor. Compared to the “Effective Planners” reference group, membership in the “Ambitious Procrastinators” profile significantly predicted higher T2 depression scores (*β* = 0.16, *p* < 0.001). Similarly, for T2 Anxiety (*β* = 0.14, *p* = 0.002) and T2 Academic Burnout (*β* = 0.20, *p* < 0.001), the “Ambitious Procrastinators” maintained the highest risk status, confirming that their mental health decline is not solely attributable to their baseline physical inactivity.

**Figure 3:**
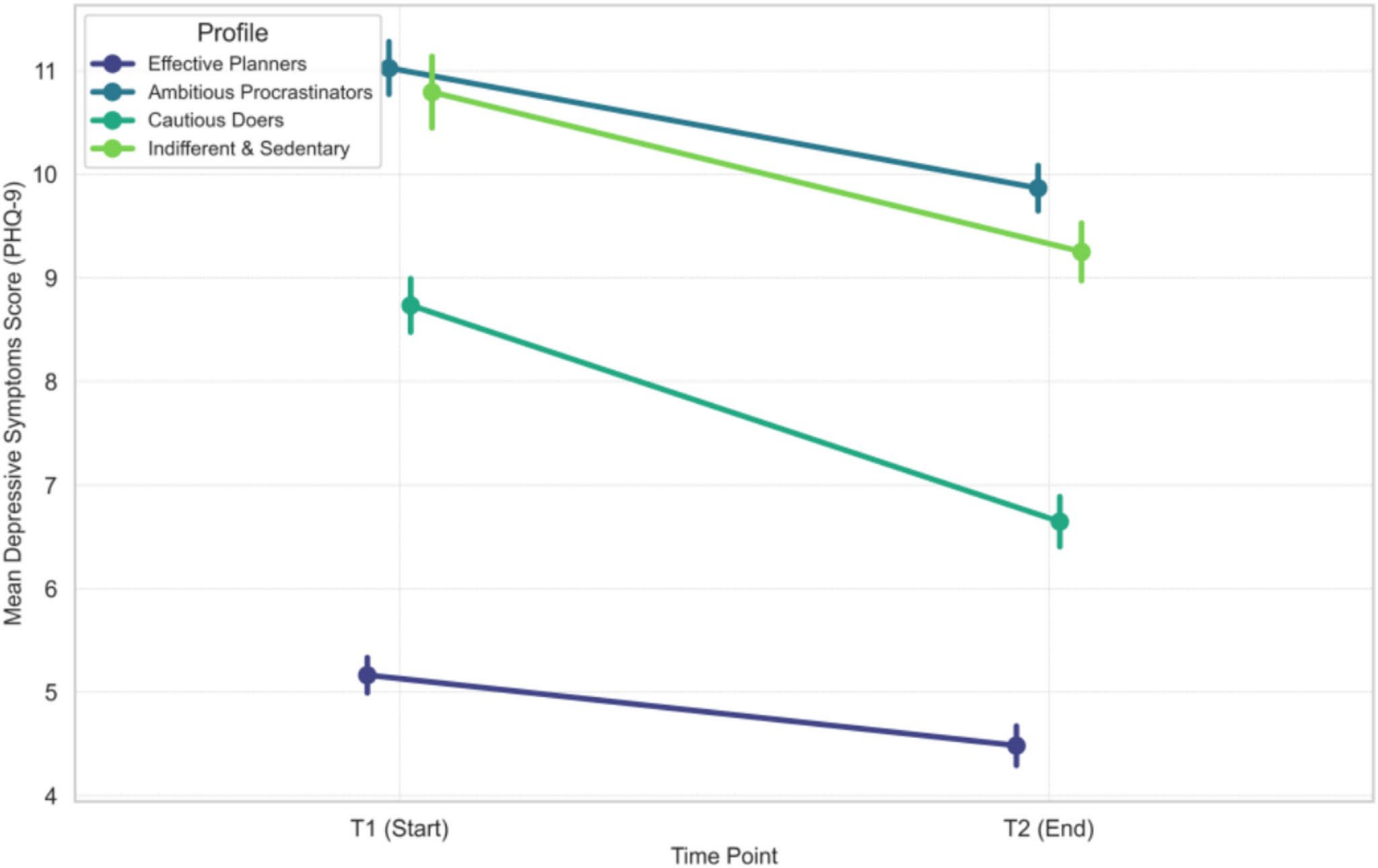
Longitudinal trajectories of depressive symptoms (PHQ-9) from T1 to T2 for each latent profile (See Supplementary material for individual spaghetti plots depicting within-group variance).

To ensure the standard errors were not biased by the “classify-analyze” approach, the sensitivity analysis using the 3-step BCH approach in Mplus corroborated these findings, demonstrating that the structural relationship between the “Ambitious Procrastinator” latent class and worsened T2 mental health trajectories remained highly significant (all p<0.01 ), firmly establishing the robustness of our results. Figure 3 shows the longitudinal trajectories of depressive symptoms, further illustrating the unique vulnerability of the “Ambitious Procrastinators.”

**Figure 4:**
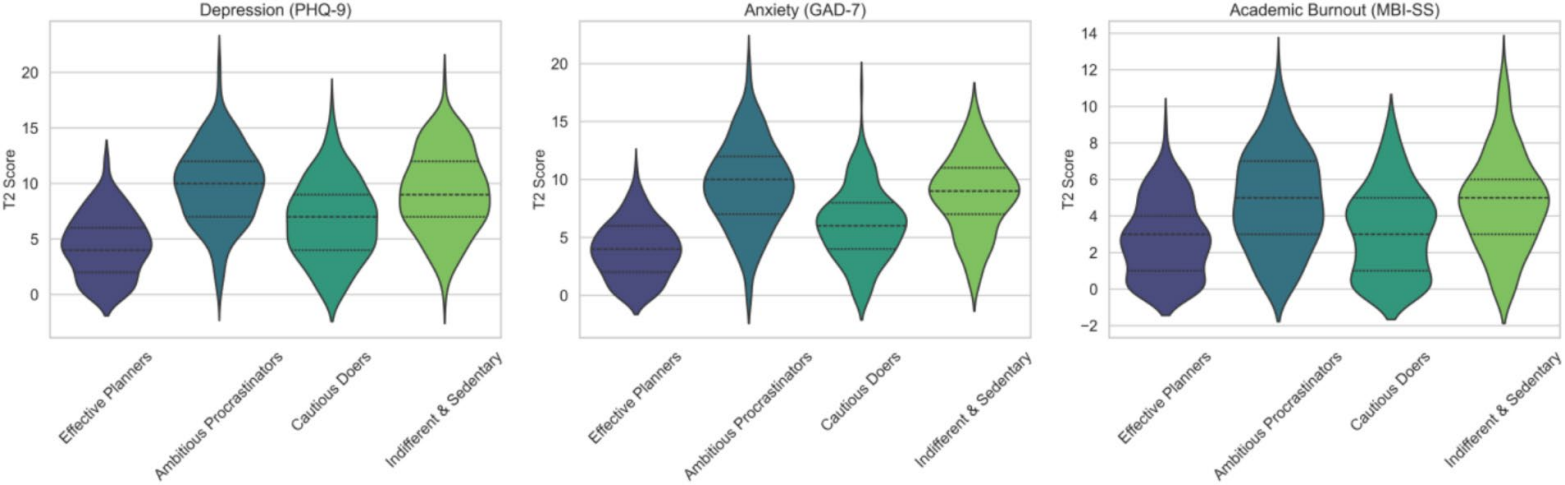
Differences in T2 mental health outcomes across latent profiles.

Finally, as vividly depicted in Figure 4, the differences in T2 mental health outcomes across profiles remain pronounced even after adjustment.

## 5 Discussion

### 5.1 Summary and interpretation of key findings

The present study employed a person-centered, longitudinal approach to investigate the psychological heterogeneity underlying the physical activity intention-behavior gap (IBG) among university students. Current findings strongly support the utility of this approach, demonstrating that the IBG is not a monolithic problem but a multifaceted phenomenon driven by distinct constellations of psychological resources and deficits. By identifying four unique profiles—"Effective Planners,” “Ambitious Procrastinators,” “Cautious Doers,” and “Indifferent & Sedentary”—the study establishes that a simple “adaptive vs. maladaptive” dichotomy is insufficient to capture the varied struggles students face in translating health intentions into action.

The most critical finding was the identification of the “Ambitious Procrastinators” as the largest subgroup (30.0%). This finding is particularly salient in the current post-pandemic context. It suggests that a significant portion of students may be experiencing a form of “re-entry shock”: possessing a heightened desire for health restoration following the global health crisis (high intention), yet lacking the renewed volitional routines to execute these goals. This profile embodies the core paradox of the intention-behavior gap: high motivation is undermined by a combination of high procrastination, perfectionistic concerns, and self-regulatory deficits. Behavioral validation revealed that their actual PA levels were statistically indistinguishable from the “Indifferent & Sedentary” group, confirming that high motivation without regulation is functionally equivalent to no motivation.

Furthermore, longitudinal results provided robust evidence for H3. Even after controlling for baseline physical activity and mental health levels, membership in the “Ambitious Procrastinators” profile remained the most robust predictor of increases in depression, anxiety, and academic burnout. This suggests that the chronic, conscious failure to meet one’s own valued health goals—a state of constant “actual-ideal” self-discrepancy—may be psychologically more taxing than simple, unconflicted apathy (Sirois and Pychyl, 2013).

### 5.2 Theoretical implications

These findings carry several important theoretical implications. First, they serve as empirical support for integrating person-centered methodologies into health behavior research. A variable-centered approach would likely have obscured the existence of the “Ambitious Procrastinators,” as the positive effect of their high intention would have been diluted by the negative effects of their volitional deficits.

Second, the present study provides a novel perspective on Self-Regulation Failure in the context of Habit Disruption. While models like M-PAC emphasize the role of habit (Rhodes, 2017), our findings highlight that in transitional periods (like the post-pandemic return), when habits are weak, the conflict between conscious planning (competence) and procrastination (liability) becomes the decisive factor. The identification of the “Ambitious Procrastinator” confirms that without the “volitional shielding” provided by habits, high intentions are easily derailed by regulatory deficits. Importantly, as campus life normalizes, habit formation research indicates that it requires consistent repetition to transition conscious effort into automaticity (Lally and Gardner, 2013). The “Ambitious Procrastinators” represent a demographic perfectly poised to benefit from structural habit-building interventions to bridge this very gap.

Additionally, locating this study within a Chinese comprehensive university context offers critical cultural insights. The profound psychological burden observed in the “Ambitious Procrastinators” is likely exacerbated by the systemic academic pressure inherent in the pervasive “involution” (Neijuan) culture. In such high-stakes environments, the pursuit of perfectionism often bleeds into health behaviors, causing students to adopt an “all-or-nothing” mentality regarding exercise, subsequently triggering anxiety-driven procrastination when ideal standards cannot be met.

Third, our longitudinal results support Self-Discrepancy Theory (Higgins, 1987) in the context of health behaviors. The finding that “Ambitious Procrastinators” experienced sharper mental health declines than the “Indifferent” group suggests that the awareness of failure is a critical mechanism. The gap between their “Ideal Self” (wanting to exercise) and “Actual Self” (not exercising) likely generates a specific form of “volitional distress,” confirming that the IBG is not just a behavioral issue, but a psychological burden.

### 5.3 Practical implications for university wellness interventions

The practical implications of these findings reject a “one-size-fits-all” approach. University wellness centers might consider a “Stratified Stepped Care Model,” where students are screened and triaged into interventions based on their psychological profile. Specifically, Tier 1 interventions should focus on Motivation Building for the “Indifferent & Sedentary” group; for these pre-intenders, evidence-based approaches such as Motivational Interviewing (MI) are best suited to resolve ambivalence.

For the critical “missing middle”—the “Ambitious Procrastinators”—Tier 2 should offer Skill-Based Cognitive Behavioral Training. Since these students are already highly motivated, further motivational messaging may be counterproductive. Instead, they require Cognitive Restructuring to dismantle the “all-or-nothing” perfectionistic mindset, Implementation Intentions to automate behavior, and specific behavioral activation strategies. For instance, incorporating the “5-min start” rule—where students commit to just 5 min of low-barrier activity to overcome initial task aversion—can systematically interrupt the procrastination loop. Finally, Tier 3 suggests recruiting “Effective Planners” as Peer Health Mentors to demonstrate effective coping planning. Regarding operational feasibility, such screening could be integrated into routine start-of-semester health assessments using brief digital scales, ensuring data privacy and cost-effectiveness.

### 5.4 Strengths, limitations, and future directions

The primary strengths of this study include its longitudinal design and its innovative use of LPA. However, several limitations must be acknowledged.

First, statistical limitations regarding the LPA must be noted. The study utilized a “classify-analyze” approach, assigning participants to profiles based on maximum posterior probabilities. While entropy was high (0.89), this method ignores classification uncertainty, which might bias standard errors in subsequent regression models. While we conducted a supplementary BCH 3-step sensitivity analysis to verify our structural parameters, future research should consistently employ 3-step approaches to better account for this latent measurement error.

Second, perfectionism and procrastination were measured using general trait scales rather than PA-specific measures. While these traits robustly predict health behaviors, domain-specific measures might yield stronger associations.

Third, a temporal mismatch exists between the PA intention measure (“next month”) and the PA behavior measure (“past week”). While common in IBG research, this limits the precision of the gap assessment. Future studies should adopt Ecological Momentary Assessment (EMA) methodologies to capture real-time, matched-interval data between localized intentions and immediate behavioral execution.

Fourth, regarding the statistical modeling, T1 PA behavior was treated as a covariate rather than an indicator in the Latent Profile Analysis. This decision was made to strictly isolate the “psychological antecedents” of the intention-behavior gap, ensuring that the profiles reflect cognitive-motivational configurations rather than behavioral habits themselves. However, we acknowledge that PA is dynamic; future research could benefit from using Latent Transition Analysis (LTA) to examine how students move between different behavioral states over time. (Note: A sensitivity analysis treating T1 PA as an indicator was evaluated and is available in the Supplemental material, showing highly consistent latent class structural validity).

Fifth, regarding generalizability, participants were recruited from a single university in China. Findings may not generalize to other institutional types or cultural contexts.

Sixth, while the longitudinal design controls for T1 baseline levels, it remains observational, and we lack T0 (university entry) baseline historical data. This limitation restricts our ability to entirely rule out pre-existing deteriorating mental health trajectories. Future research should leverage Latent Growth Curve Modeling (LGCM) over extended timelines to track full developmental trajectories. Furthermore, unmeasured confounding variables (e.g., executive function deficits, chronic sleep quality deterioration, and cumulative academic stress) cannot be fully ruled out.

Future directions should focus on conducting a Randomized Controlled Trial (RCT) assigning “Ambitious Procrastinators” to the specific CBT-based skill-building intervention proposed above to test the causal mechanism of change.

### 5.5 Conclusion

The present study provides robust, longitudinal evidence that the struggle to translate physical activity intentions into action is not a uniform problem. In the post-pandemic era, the identification of the “Ambitious Procrastinator”—a large, highly motivated yet ineffectual group—is a critical finding. This profile is not only at risk of failing to achieve health goals but is also uniquely vulnerable to a longitudinal decline in mental health. University wellness initiatives must evolve from generic motivational campaigns to precision-based, skill-building programs targeting the specific self-regulatory needs of these students.

## Data Availability

The raw data supporting the conclusions of this article will be made available by the authors, without undue reservation.
