# Supplementary material for: When high hopes meet low action: identifying the “Ambitious Procrastinator” profile in the physical activity intention-behavior gap and its mental health toll
Source: Front Psychol. 2026 Mar 30;17:1804409. doi: 10.3389/fpsyg.2026.1804409 (PMC13071067; doi:10.3389/fpsyg.2026.1804409)
Supplement: Supplementary file 1 [file Table_1.docx]

Table S1. Regression Coefficients for Demographic Covariates Predicting T2 Mental Health Outcomes

| **Outcome & Predictor** | **B** | **SE** | **β** | **p** |
| --- | --- | --- | --- | --- |
| **Model 1: T2 Depression (PHQ-9)** |  |  |  |  |
| Constant | 1.12 | 0.45 |  | .013 |
| Age | -0.05 | 0.04 | -.03 | .215 |
| Gender (1 = Male) | -0.32 | 0.18 | -.04 | .078 |
| Academic Year | 0.15 | 0.10 | .03 | .135 |
| **Model 2: T2 Anxiety (GAD-7)** |  |  |  |  |
| Constant | 0.85 | 0.41 |  | .038 |
| Age | -0.02 | 0.03 | -.02 | .542 |
| Gender (1 = Male) | -0.45 | 0.15 | -.06 | .003 |
| Academic Year | 0.08 | 0.09 | .02 | .371 |
| **Model 3: T2 Academic Burnout (SBI)** |  |  |  |  |
| Constant | 0.42 | 0.35 |  | .230 |
| Age | 0.01 | 0.02 | .01 | .765 |
| Gender (1 = Male) | -0.08 | 0.12 | -.02 | .490 |
| Academic Year | 0.22 | 0.06 | .11 | <.001 |

Note. This table provides the full coefficient data for the demographic covariates (Age, Gender, Academic Year) that were summarized in the main text Table 4. B = Unstandardized coefficient; SE = Standard Error; β = Standardized coefficient.
